# Making every test count: Insights from a test kit efficiency audit

**DOI:** 10.4102/ajlm.v15i1.3013

**Published:** 2026-07-23

**Authors:** Amy O. Strydom, Mpho R. Maphayi, Chemedzai Chikomba

**Affiliations:** 1 School of Pathology, Department of Chemical Pathology, Faculty of Health Sciences, University of the Witwatersrand, Johannesburg, South Africa; 2 Department of Chemical Pathology, National Health Laboratory Services, Charlotte Maxeke Johannesburg Academic Hospital, Johannesburg, South Africa

**Keywords:** audit, test kit efficiency, lean management, laboratories, automated laboratories

## Abstract

**Background:**

Clinical laboratories face increasing demand for testing while operating within constrained budgets, necessitating efficient resource utilisation. Test kit efficiency, a key parameter in automated laboratories, refers to the appropriate use of reagent kits in a manner that minimises non-billable tests per kit.

**Intervention:**

Manufacturer-supplied monthly test kit efficiency data for 96 chemistry analytes, collected between 01 April and 30 September 2023, were assessed retrospectively and compared with their respective benchmark efficiencies. The findings were further compared to those from a previous audit to assess for an improvement or decline in efficiency. Under-performing analytes identified included total protein in cerebrospinal fluid, alkaline phosphatase, glucose, ferritin, direct bilirubin and vancomycin. Root cause analysis and targeted interventions led to improvements in test kit efficiency of 12.6% – 37.7% for each analyte.

**Lessons learnt:**

Auditing test kit efficiency data revealed inefficiencies, allowing targeted interventions to optimise kit use and reduce costs without compromising quality. Efficiency was influenced by analytical performance, test kit size and batching practices, underscoring the importance of data-driven decision-making for workflow optimisation and resource management. The audit revealed challenges with inherently inefficient tests, highlighting the need for tailored solutions based on the population served, reagent stability and sample stability.

**Recommendations:**

We recommend regular test kit efficiency audits to reduce wastage, optimise workflow and drive quality improvement. Efficiency data can support identification of problematic assays and guide kit size adjustment and batching strategies, while ensuring initiatives remain patient-centred. However, the lack of standardised regulatory guidelines on test kit utilisation remains a key limitation that requires attention.

**What this study adds:**

There is limited published evidence on the use of test kit efficiency data to drive quality improvement in clinical chemistry laboratories. This study demonstrates how systematic auditing can identify modifiable inefficiencies and support evidence-based resource optimisation.

## Background

Across the globe, healthcare systems are under continuous pressure to reduce costs while maintaining high standards of quality.^1^ Clinical laboratories are not exempt, as they face increasing demand and test utilisation, while budgets often do not scale to meet these growing needs.^1,2^

Several drivers of increased laboratory utilisation have been proposed.^2,3,4^ These include factors such as the growing geriatric population, the rising number of patients living with chronic diseases, the expanded range of testing options available owing to technological advancements and defensive test-ordering practices driven by litigation concerns, among others.^2,3,4^ Collectively, these factors place additional strain on laboratory resources, necessitating innovative approaches to manage demand and optimise efficiency.

Buches et al. define efficiency as a parameter that assesses a system’s ability to achieve higher levels of performance relative to the inputs consumed.^3^ Consequently, sources of inefficiency or waste in the laboratory system should be identified and minimised where possible. Lean management principles, which focus on reducing waste and optimising workflows, are integral to this approach.^5^ By streamlining processes and optimising resource utilisation, lean techniques enable laboratories to improve performance and efficiency without compromising quality.^5^ A key parameter of the analytical phase of the total testing process that can be assessed and improved in laboratories is test kit efficiency.

Reagent test kits are routinely used in many automated laboratory systems.^6^ Each analyte requires a specific reagent test kit, containing a defined volume of reagent sufficient for a predetermined number of tests.^6^ Test kit efficiency refers to the appropriate use of reagent kits in a manner that maintains adequate output (i.e. number of billable tests analysed) while limiting test wastage. Factors that reduce test kit efficiency, whether avoidable or unavoidable, can be categorised as non-billable or discarded tests (Box 1).

BOX 1 Factors that decrease test kit efficiency.

**Non-billable tests**

- Tests used for assay calibrations
- Tests used as part of internal quality control (IQC)
- Tests used for troubleshooting of out-of-control IQC
- Tests used for external quality assurance (EQA)
- Tests used for confirmation of unusual or unexpected results
- Tests used for re-analysis to confirm tube additive contamination (e.g. calcium and alkaline phosphatase [ALP] testing to confirm ethylenediaminetetraacetic acid [EDTA] contamination in samples with spuriously high potassium concentrations)
- Tests used to perform dilutions
- Tests used for inter-instrument comparisons or patient correlation studies

**Discarded tests**

- Remaining tests from test kits that have expired
- Remaining tests from test kits that have exceeded on-board stability due to kit volumes being too large for the number of tests requested
- Remaining tests from test kits with short on-board stability
- Remaining tests displaying poor IQC performance as reagent volume becomes low in the test kit

There is currently no standardised approach to calculating test kit efficiency. The method described below reflects the approach used in our laboratory at the time of the audit. Test kit efficiency data are received from the manufacturer monthly and include the following parameters for each analyte: the total number of tests available on the kit, the number of billable tests, the number of tests used for calibration, the number of tests used for internal quality control (IQC) analysis, the number of repeat analyses (or ‘reruns’) and the number of discarded tests or ‘wastage’.^7^ The monthly percentage test kit efficiency is then calculated by the manufacturer using the formula^7^ in Equation 1:


$$\begin{array}{l} \%\text{ test kit efficiency}=\text{billable tests}÷(\text{number of tests}\\ \text{ consumed}+\text{wastage})\times 100 \end{array}$$ [Eqn 1]

The number of billable tests for each analyte is calculated by the manufacturer by subtracting the total number of tests used for calibrations, controls and repeat analyses from the total number of tests analysed for that month.^7^ Therefore, the assumption is made that a test is billable as long as it is not used for calibrations, controls or repeats. The number of tests consumed per month includes the number of billable tests, tests used for calibrations, tests used for IQC and tests used for repeat analyses, while wastage includes any discarded tests.

Each analyte’s test kit efficiency is compared to its respective benchmark efficiency. In an automated clinical chemistry laboratory, the benchmark is a dynamic, predefined target value that represents the expected efficiency of test kits for a given analyte, accounting for routine operational factors. As no standardised approach for determining benchmark efficiencies exists, these must be established by the laboratory or the manufacturer for each analyte. In our laboratory, the benchmark efficiency (%) is determined by the manufacturer and is calculated from the number of billable tests for that month, together with an assumed number of tests required for reruns, IQC and routine calibrations. These assumptions are based on consensus laboratory data and may be adjusted according to local experience. As such, the benchmark efficiency varies from month to month.

By comparing an analyte’s actual test kit efficiency (derived from real-world usage) to its benchmark efficiency, a laboratory can determine whether tests kits are being used adequately (i.e. whether the actual efficiency meets or exceeds its benchmark efficiency) or whether inefficiencies in test kit usage are present (e.g. from excessive reruns owing to problematic IQC and troubleshooting, or wastage owing to kit expiry and improper handling).

Monitoring the efficiency of test kits is essential for ensuring optimal laboratory performance, controlling costs and supporting continuous quality improvement. This process provides valuable insights into operational inefficiencies, enabling identification of waste and streamlining of workflows. By systematically assessing test kit utilisation, laboratories can reduce unnecessary expenditure, improve resource allocation, and enhance overall diagnostic service delivery.

This report discusses the key lessons learnt from audits of test kit efficiency and the mitigation strategies implemented at the National Health Laboratory Service automated chemical pathology laboratory at Charlotte Maxeke Johannesburg Academic Hospital (CMJAH) in Johannesburg, South Africa. The findings underscore the importance of ongoing monitoring and audit-driven interventions in optimising laboratory processes, ensuring sustainability, and enhancing patient care.

## Description of the intervention

### Audit design

This was a retrospective, observational study based on secondary data analysis and did not involve direct patient data. Test kit efficiency data for 96 clinical chemistry analytes were collected over a 6-month period (01 April to 30 September 2023) from the automated chemical pathology laboratory at CMJAH, an ISO 15189-accredited facility. The laboratory submitted raw instrument performance data to the manufacturer (Roche Diagnostics^®^), who processed the data and returned the calculated test kit efficiency for review as part of their standard service. No internal analysis of the data was performed prior to submission to the manufacturer.

To evaluate trends in efficiency over time, the 2023 efficiency metrics were compared to results from a test kit efficiency audit conducted in 2022, allowing assessment of changes in performance patterns and potential improvements. Each analyte that did not achieve an efficiency above its benchmark was examined individually to determine and address the specific root cause of the inefficiency.

#### Key performance metrics

The following metrics, as reported by the manufacturer, were reviewed:

- Billable tests – the total number of completed and chargeable tests performed per month per analyte (calculated by subtracting the total number of tests used for calibrations, IQC and repeat analyses from the total number of tests analysed).
- Calibrations – the number of tests used for calibration per month for the analyte (including routine calibrations and calibrations performed as part of troubleshooting).
- Internal quality control analyses – the number of tests used for IQC runs per analyte, including IQC runs used routinely or as part of troubleshooting.
- Reruns – the number of repeated tests performed on a sample for a given analyte because of dilutions, middleware rules, inter-instrument comparisons, instrument errors or the need to verify unusual or unexpected results. Any test flagged by the analyser as having been run more than once is included in this metric.
- Wastage – the number of tests discarded owing to the reasons listed in Box 1 under ‘Discarded tests’.

### Ethical considerations

An ethics waiver was obtained from the University of the Witwatersrand Health Research Ethics Committee (W-PR-241127-02) as the study involved no humans or animal subjects.

## Results

### Overall findings

**FIGURE 1:**
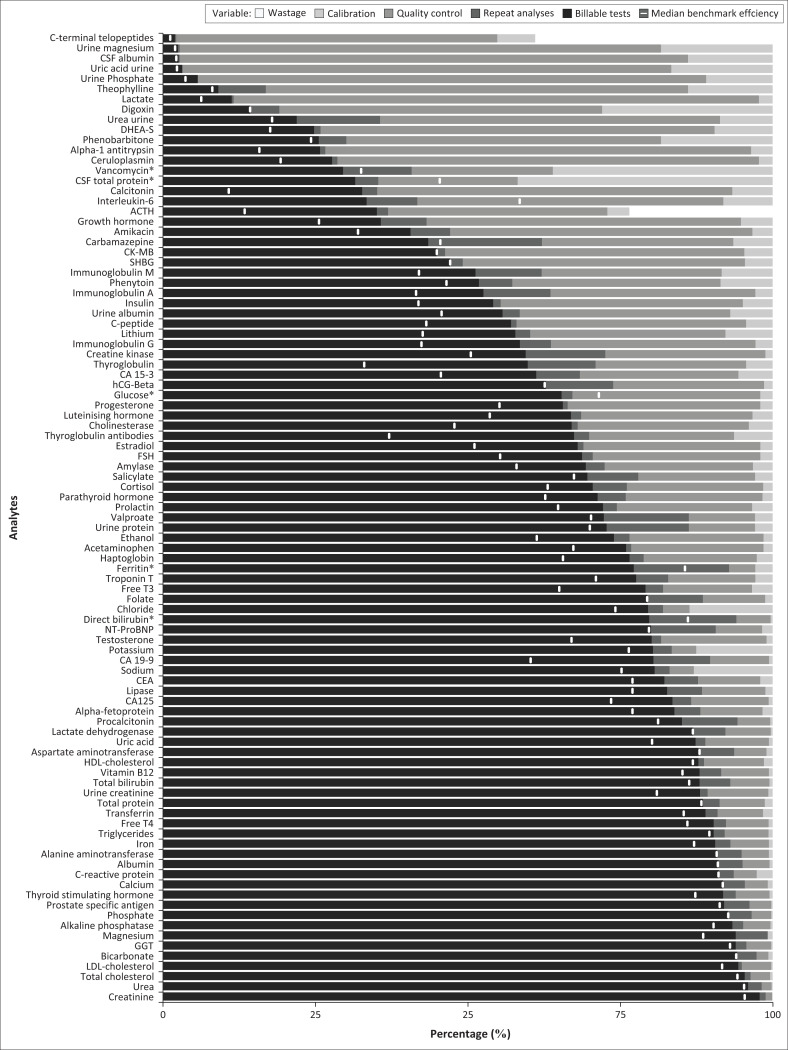
Stacked bar chart illustrating the median percentage of total tests consumed by each efficiency-related variable per analyte, based on monthly data from the 2023 audit period. Analytes not meeting their respective benchmarks are indicated with an * on the *y*-axis.

For the 2023 audit, 91 of the 96 analytes (94.8%) achieved adequate test kit efficiency compared to their benchmark efficiencies. A breakdown of the audited data is included in Figure 1. Of the five analytes with test kit efficiencies below their respective benchmarks, one had previously achieved adequate efficiency, whereas the remaining four had been persistently problematic since the 2022 audit. In the 2022 audit, 89 out of the 96 analytes (92.7%) achieved adequate efficiency compared to their respective benchmarks. Of the seven analytes that did not achieve adequate efficiencies, three showed improved efficiency after the suggested interventions were implemented.

### Problematic assay review

In the following sections, we present an analysis for the problematic analytes identified in both audit periods (Table 1). We outline the cause of poor test kit efficiency performance, the corresponding mitigation strategies implemented, and the improvement noted post-implementation.

**TABLE 1:** Test kit and benchmark efficiency data (2022 and 2023 audit) for analytes with poor performance.

Analytes	2022 Audit efficiencies				2023 Audit efficiencies			
	Test kit efficiency (%)		Benchmark efficiency (%)		Test kit efficiency (%)		Benchmark efficiency (%)	
	Median %	Interquartile range	Median %	Interquartile range	Median %	Interquartile range	Median %	Interquartile range
TP-CSF	51.9	48.2–53.3	36.9	33.1–42.2	31.8	31.2–31.85	45.4	44.6–45.5
ALP	61.0	56.9–63.0	90.6	90.4–90.7	93.5	93.0–93.6	90.3	90.2–90.35
Glucose	30.0	29.9–33.7	71.0	70.8–73.3	66.4	65.7–68.1	71.5	71.0–73.25
Ferritin	72.0	68.3–77.4	83.6	82.6–85.0	76.9	75.0–78.3	85.6	85.4–85.8
Direct bilirubin	77.7	76.7–78.2	84.3	83.5–84.8	78.9	78.6–79.4	86.1	85.6–86.3
Vancomycin	34.2	28.1–36.7	43.3	40.5–51.2	25.0	21.5–25.8	32.5	32.0–35.6

TP-CSF, total protein in cerebrospinal fluid; ALP, alkaline phosphatase.

All data are presented as median %.

The interquartile range is included within the square brackets.

#### Total protein in cerebrospinal fluid

**FIGURE 2:**
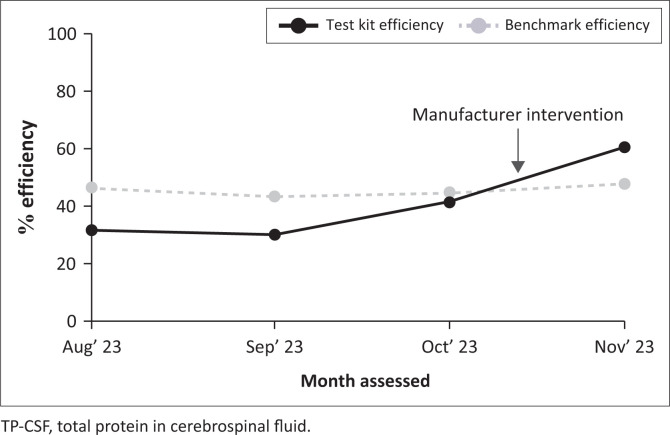
Total protein in cerebrospinal fluid test kit efficiency data from August to November 2023.

Total protein in cerebrospinal fluid (TP-CSF) was noted to have poor test kit efficiency across the months of August 2023 and September 2023 with test kit efficiencies of 31.8% (benchmark 46.7%) and 30.6% (benchmark 43.8%). It was found that the number of tests used for IQC analyses and calibrations was higher than expected for those months, explained by multiple IQC failures and calibration signal errors. Manufacturer-led troubleshooting found several possible hardware issues that may have been the cause of poor analytical performance. Once these issues were addressed by the manufacturer, the calibration of the TP-CSF assay was successful. The reduction in tests used for repeated unsuccessful calibrations and the improved IQC performance of the TP-CSF assay led to improved efficiencies in November 2023 of 60.4% (benchmark 47.8%) (Figure 2).

#### Glucose and alkaline phosphatase

In the 2022 efficiency audit, glucose was noted to have a median test kit efficiency of 30.0% (benchmark 71.0%). Similarly, alkaline phosphatase (ALP) also showed poor test kit efficiency, with a median efficiency of 61.0% (benchmark 90.6%). Investigation revealed a high number of discarded tests with a median monthly wastage of 1347 and 2691 tests for glucose and ALP. This occurred because the reagent kits were oversized relative to test request volumes and the on-board stability period for each analyte. Once the test kit sizes were reduced for glucose (2200 tests per kit to 600) and ALP (1050 tests per kit to 400) the number of discarded tests was reduced to zero, leading to an improvement in test kit efficiency of 67.7% (benchmark 72.5%) and 92.3% (benchmark 90.2%).

During the 2023 audit period, the test kit efficiencies for both glucose and ALP remained stable, with median efficiency values of 66.4% (benchmark 75.6%) and 93.5% (benchmark 90.3%). The persistently reduced efficiency observed for glucose was attributable to increased consumption of tests for IQC. The aforementioned TP-CSF analytical issues necessitated offering this test on two instruments in the laboratory to allow for troubleshooting on the problematic instrument without negatively affecting laboratory operations. To maintain analytical workflow, glucose (including glucose-CSF and plasma glucose, which utilise the same reagent kit) was also analysed on the second instrument, as these analytes are frequently co-requested. This change resulted in at least a twofold increase in tests consumed for IQC and routine calibrations. As test request volumes remained largely unchanged, glucose test kit efficiency persistently performed below the benchmark throughout the audit period. Test kit efficiency data for subsequent months were unavailable because the contract with Roche Diagnostics^®^ ended and a new supplier was selected. Therefore, any potential improvements following the consolidation of glucose analysis to a single instrument could not be assessed.

#### Direct bilirubin

**FIGURE 3:**
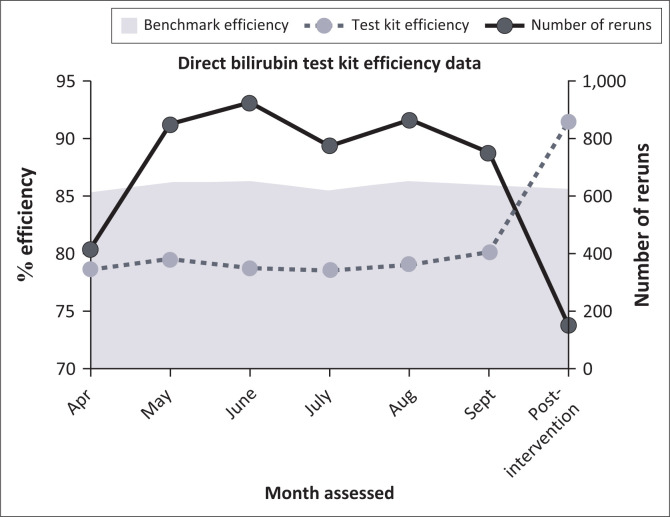
Bilirubin test kit efficiency pre-intervention and post-intervention.

Direct bilirubin demonstrated consistently poor test kit efficiencies (Figure 3) owing to a high number of rerun tests, with a median 775 rerun tests monthly (17.9% of the median 4324 billable tests). Upon investigation, it was noted that a rule was programmed on the laboratory middleware to automatically reanalyse direct bilirubin samples if the concentration was lower than the lower limit of quantitation for the assay (2 µmol/L). Considering that many patients have direct bilirubin concentrations below the lower limit of quantitation (reference interval as per the laboratory information system: 0 µmol/L – 3 µmol/L), this rule was removed from the middleware.^8^

Analytical errors in direct bilirubin measurement can be detected by other means, including the correlation of results with other liver function parameters (e.g. total bilirubin, ALP, gamma-glutamyl transferase), evaluation for discrepancies between the direct bilirubin and total bilirubin result, as well as with the icterus index, which is known to correlate with hyperbilirubinaemia.^9^ Following removal of the rule, direct bilirubin reruns decreased to 145 tests (3.6% of 4021 billable tests) in the subsequent month, increasing test kit efficiency to 91.5% (benchmark 85.6%).

#### Ferritin

Test kit efficiencies fell below the benchmark throughout the audit period for ferritin (Table 2). It was found that the assay consistently produced multiple calibration signal errors that persisted despite troubleshooting. The Roche^®^ immunoturbidimetric assay for ferritin measurement requires a six-point calibration, utilising 12 tests per calibration; consequently, multiple failed calibrations resulted in poor test kit efficiency. It was evident that in months in which fewer tests were used for calibration, there was an improvement in efficiency, while the months with higher numbers of tests used for calibrations (April 2023 and September 2023) had the lowest efficiencies for the audit period. Notably, in April and September more tests were consumed for calibrations than for IQC, providing clear evidence of a recurrent calibration problem. To the best of our knowledge, there has been no formal communication from the manufacturer regarding calibration failures for the fourth generation immunoturbidimetric ferritin assay.

**TABLE 2:** Test kit efficiency data for ferritin.

Parameter	April 2023	May 2023	June 2023	July 2023	August 2023	September 2023
Efficiency %	71.6	78.7	76.9	76.9	79.1	74.4
Benchmark %	83.9	85.6	86.0	85.3	85.6	85.9
Billable tests	2313.0	2768.0	2977.0	2687.0	2785.0	2984.0
Reruns	558.0	584.0	583.0	568.0	520.0	528.0
Calibration	192.0	26.0	109.0	92.0	78.0	270.0
Controls	167.0	140.0	202.0	145.0	138.0	227.0

Despite the wide analytical range of the ferritin assay (5 µg/L – 1000 µg/L, which can be extended to 8000 µg/L following a 1:8 dilution) a large proportion of tests were used for reruns (mainly for dilutions of results above the upper limits of the measuring range) which also decreased test kit efficiency. However, because ferritin has utility as a prognostic biomarker in malignancy and critical care,^10,11,12^ tests used for dilutions to provide an absolute value are justified. The high proportion of reruns (median 20.3% of billable tests) may reflect the unique patient population served by CMJAH, a quaternary centre with a substantial oncology and rheumatological case load and a high prevalence of HIV and tuberculosis infection.^13^ In addition, during the audit period, the coronavirus disease 2019 pandemic may have further increased demand for ferritin testing.

#### Vancomycin

The test kit efficiencies of vancomycin for April to August 2023 reflected the IQC and calibration issues of the assay with a median efficiency of 25.0% (benchmark 32.5%). After troubleshooting the vancomycin assay IQC performance, the manufacturer escalated the issue to their global support team. The testing for this analyte was moved to the Roche Integra^®^ analyser to ensure continued service. The Roche Integra^®^ analyser did not allow for data extraction for efficiency calculations and thus the efficiency after the intervention could not be compared. Despite the issues with IQC performance for vancomycin, EQA performance remained acceptable throughout the audit period, but the poor IQC and test kit efficiency performance required relocation of testing to another instrument.

## Lessons learnt

Ensuring efficient utilisation of test kits is a critical component of laboratory resource management, balancing cost-effectiveness with quality assurance. This audit highlighted key inefficiencies in test kit usage and the impact of analytical performance on test kit efficiency, as well as the important role that targeted interventions played in improving efficiency. By systematically assessing test kit efficiency across 96 analytes, we identified opportunities for improvement and implemented corrective actions that improved test kit performance. These findings underscore the importance of data-driven decision-making in laboratory operations. Importantly, the audit provided valuable lessons that can guide future efficiency assessments and inform best practices in test kit management. A summary of the effect of specific interventions on test kit efficiency is presented in Table 3.

**TABLE 3:** Analytes that were amenable to intervention and their respective pre- and post-intervention efficiencies.

Analyte	Intervention	Test kit efficiency (%)	
		Pre-intervention	Post-intervention
TP-CSF	Hardware intervention	31.8	60.4
ALP	Reduction kit size	61.0	92.3
Glucose	Reduction kit size	30.0	67.7
Direct bilirubin	Optimisation of middleware rules	78.9	91.5

TP-CSF, total protein in cerebrospinal fluid; ALP, alkaline phosphatase.

Lessons learnt following the test kit efficiency audits included the following:

**Identification of inefficiencies through audit:** Auditing test kit efficiency allowed the laboratory team to identify sources of inefficiency and implement strategies to address them, reducing costs without compromising the standard of patient care.

**The impact of analytical performance on test kit efficiency:** Analytical challenges reduced test kit efficiency, as seen for the TP-CSF, ferritin and vancomycin assays. Addressing these technical problems through troubleshooting and manufacturer intervention resulted in an improvement in efficiency.

**Test kit size and test kit efficiency:** Test kit efficiency data provided the quantitative evidence required for decision-making about test kit size adjustments, as seen with glucose and ALP. Test kit size needed to be considered in conjunction with the test request volumes expected and the on-board stability of the reagent.

**Test-batching and optimising internal quality control frequency:** While not an objective of this audit, we also noted that some analytes, although not flagged as having a test kit efficiency below benchmark, had low efficiencies owing to having low test request volumes. An approach to improving test kit efficiency in such analytes may include batched analysis when appropriate. By decreasing the number of tests used for IQC per billable test, test kit efficiency could be improved.

**Challenges with specific tests:** Despite interventions, some analytes remained inefficient owing to non-modifiable limitations, such as a lack of smaller reagent kits or low test demand. For such analytes, the clinical needs of the patient population being served, guided by local practice standards and the potential impact on patient management, may justify maintaining certain tests on the test menu, despite the potential financial loss incurred. At large sites, losses from tests with inherently poor efficiency may be partially offset by high-volume, high-demand tests (e.g. urea, creatinine, electrolytes). Referring low demand tests to other laboratories may also be considered.

**The need for standardisation in test kit efficiency evaluation:** The absence of standardised methods for assessing test kit efficiency posed challenges when the laboratory changed suppliers. Variations in the data provided by different manufacturers prevented meaningful comparison of performance and limited continuation of post-intervention assessment. This audit highlighted the importance of developing standardised approaches for test kit efficiency evaluation.

**The role of artificial intelligence in laboratory workflow optimisation:** Artificial intelligence is increasingly recognised as a powerful tool for optimising laboratory workflow efficiency. Although our laboratory has not yet implemented artificial intelligence-driven systems, international experience demonstrates that machine-learning tools can improve stock management, anticipate testing demand and detect workflow inefficiencies that contribute to excess reagent use.^14,15,16^ Artificial intelligence-enabled middleware and automation platforms have also been shown to streamline sample routing, reflex testing and instrument scheduling.^14,15,16^ Integrating these systems into routine operations may enhance test kit utilisation, strengthen value-driven laboratory practice and allow real-time rather than retrospective assessment.

## Strengths and limitations

This audit had several strengths. It included a comprehensive assessment of all 96 clinical chemistry analytes offered in the automated laboratory at CMJAH. Two audits were conducted, allowing continued monitoring of test kit efficiency over time and comparison of performance trends. Furthermore, this report contributes to the largely unexplored topic of test kit efficiency, addressing a gap in the current literature.

There were several limitations. At present, no regulatory body provides standardised guidance on how to assess test kit efficiency or on how to establish benchmark efficiencies for comparison. The formula currently in use in our laboratory is inherently limited, as it assumes that all analysed tests are billable unless they are allocated to calibrations, controls or reruns. As a result, it does not account for other sources of non-billable tests such as those used for EQA and contamination checks. Because the manufacturer was unable to provide quantitative data for all potential sources of non-billable tests, it was not possible to determine the full extent to which these factors may have affected the total number of billable tests. However, EQA samples contribute to less than 0.5% of tests per kit and are therefore not expected to have a meaningful impact on the number of billable tests or efficiency levels. Lastly, post-intervention data were limited owing to a change in laboratory supplier following the audits, reducing the ability to assess the impact of workflow modifications.

## Recommendations

The general recommendations following this audit include:

- Perform regular test kit efficiency audits to improve laboratory efficiency in the analytical phase of testing.
- Use test kit efficiency data to justify adjustment of kit sizes for analytes with low test request volumes and high wastage.
- Incorporate use of test kit efficiency data as an additional quality measure to identify analytes with poor analytical performance.
- Apply test kit efficiency data to support the batching of suitable tests, taking into consideration the test request volumes, clinical utility, turnaround time, reagent on-board stability and sample stability.
- Optimise laboratory workflow based on test demand to minimise non-billable tests.
- Balance efficiency initiatives with the laboratory’s role in patient care, ensuring value-based management remains the core aim of the laboratory.
- Integrate validated artificial intelligence-driven systems into laboratory operations to improve test kit utilisation, streamline workflow processes and support data-driven operational decisions.
- We advocate for regulatory bodies to provide standardised guidelines for test kit efficiency and benchmark establishment to enable meaningful comparisons between manufacturers and laboratories.
